# Wasted Holidays

**Published:** 1912-08-17

**Authors:** 


### WASTED HOLIDAYS.

At this time of the year the majority of the professional classes and hospital workers generally are *en famille* at the seaside or in the country. Both certainly hope to leave behind them all thoughts of medicine, whether as drugs to be taken or as a science to be studied and applied, and it seems almost unkind to remind them that, though the best holidays are the least carefully planned, holiday-making, too, has its principles, without which it will become, as many in fact find it, a period of disturbance and anxiety, the end of which is a positive relief. Much has been written of holidays and the way in which to spend them, and every form of recreation from life in a living-waggon to a trip to Dieppe has been held up as the best method. With these suggestions we have nothing to do; those engaged in institutional life will be the best judges. But now that the holiday season is in full swing we may answer the dumb inquiries of those who are perhaps feeling their own to be disappointing. Holidays are simply the periodic demand of the living organism for change of environment; men and women are not machines, but, like a bridge traversed by a regiment, will break if the uniform tread of events is never broken in its rhythm.

This uniformity is particularly felt in institutional life, and those engaged in it should be particularly careful to secure the change which they need. Very rarely do they do so. For the way of institutional life invites to matrimony, and the ranks of institutional workers are filled with married men. The question then arises, Should holidays be spent *en famille?* Sentiment answers Yes; common sense and science reply in the negative. For, as a general rule, it must be laid down that to transport a family to the seaside or to a house in the country does not break the monotony of life. Recreation, in its strict sense of regeneration, is not usually possible on holidays *en famille*. The opening of the holiday, the search for a house, the transport of children and much luggage, these have led quite naturally many a married man to sigh: "With my staff and scrip once I passed over Jordan; but now I am become two bands." And what is often a worry to the head of the family is bad also for the women and children. With the women's case we have not space to deal; but no wife will need reminding that a holiday which entails no rest from housekeeping is at once open to very severe criticism from the woman's point of view. As Shakespeare knew, men are "merriest when they are from home." The man therefore, as a general rule, should be a bachelor again at this season and spend it away from the cares that are rightly the concomitants of happy family life. That is the first principle of change; it is, indeed, the only great change which a married man can make, and which is naturally impossible, if not entirely undesired, at any other season. This principle granted, he may spend his time where he will, and if his days and nights be tinged with regret for his voluntarily enforced isolation from family ties, he will gladly suffer this as knowing that his regret is the sensible proof that his holiday has involved a real change, and that in its deepest sense it has not been wasted. He will embrace routine again with a new vigour, from which both his family and his institution will be the first to gain.

In another part of this issue the "institutional atmosphere" which at its worst turns men into shallow routineers is examined, and the way in which Poor-Law Infirmary officers may escape it is described. That atmosphere, however, in a less degree is the inevitable risk attendant on institutional life in all its forms, and that is why hospital workers more, probably, than most men should see to it that their holidays are not wasted. It is not that men and women, least of all those in hospital life, should take their pleasures sadly; it is that they should recognise how much more they need a real change in those often too short periods when the hospital, the infirmary, or the home is to be left behind. A light heart is a bachelor's perquisite, for married men needs must give hostages to fortune in return for the privileges of their state. But a light heart is the best companion on a holiday.

